# Revealing neurovascular coupling at a high spatial and temporal resolution in the living human retina

**DOI:** 10.1126/sciadv.adx2941

**Published:** 2025-06-27

**Authors:** Pierre Senée, Léa Krafft, Inès Loukili, Daniela Castro Farias, Olivier Thouvenin, Michael Atlan, Michel Paques, Serge Meimon, Pedro Mecê

**Affiliations:** ^1^ Institut Langevin, ESPCI Paris, CNRS, PSL University, Paris, France.; ^2^ DOTA, ONERA, Université Paris Saclay, F-91123 Palaiseau, France.; ^3^ Institut de la Vision, Sorbonne Université, INSERM, CNRS, 17 rue Moreau, F-75012 Paris, France.; ^4^ Paris Eye Imaging Group, Centre d’Investigation Clinique 1423, Quinze-Vingts Hospital, INSERM, Paris, France.

## Abstract

Neurovascular coupling (NVC) regulates local blood flow in response to neuronal activity, yet its precise characterization at the capillary level has been hindered by the lack of a noninvasive, high-resolution imaging method. Here, we introduce the adaptive optics rolling slit ophthalmoscope, a unique noninvasive, label-free, high-speed, cellular-resolution clinical imaging system for assessing retinal NVC in the living human eye. Using an off-axis phase contrast approach combined with camera-based confocal slit gating, our method provides large field-of-view imaging of arterial and venular walls, enabling the study of vascular dynamics with unprecedented spatiotemporal precision. Our findings highlight that this level of precision is essential for accurately distinguishing NVC-driven vasodilation from spontaneous fluctuations, such as vasomotion and the cardiac cycle. By bridging the gap between fundamental neurovascular research and clinical applications, this approach offers a powerful tool for neuroscience research and early disease detection and monitoring of neurodegenerative and vascular disorders.

## Teaser

An optical method reveals neurovascular coupling in the human retina with unprecedented spatial and temporal resolution.

## INTRODUCTION

Upon activation of specific brain regions, a localized augmentation in cerebral blood flow ensues, facilitating the delivery of oxygen and essential nutrients to meet the increased metabolic demands due to neural activity. This physiological response is known as neurovascular coupling (NVC). First reported over a century ago by Roy and Sherrington (*1*), NVC has since been a key driver of extensive brain research (*2*, *3*) where it is the source of the BOLD (blood oxygenation level dependent) signal detected with functional magnetic resonance imaging (fMRI) (*4*).

Dysregulation of NVC is at the core of several neuropathological conditions, including ischemic stroke (*5*), Alzheimer’s disease (*6*), and cognitive impairments stemming from hypertension (*6*). Therefore, a precise characterization, in space and time, of the cellular mechanisms underlying physiological and/or pathophysiological NVC responses can facilitate current queries in neurovascular metabolism research (*7*–*9*). Moreover, such precise characterization can provide biomarkers for the evaluation of these diseases and may ultimately lead to the development of therapies to prevent the breakdown of NVC in their treatment (*7*–*9*). However, current analysis of NVC in the human brain is limited by millimeter-resolution techniques, such as fMRI (*10*, *11*). To achieve micrometer cellular resolution, one has to go to optical wavelengths. Unfortunately, because of light scattering in cranial and brain tissues, such micrometer resolution is accessible only in animal studies after a surgical exposure of the cortex (*12*, *13*).

A promising path is to use the eye as a window to the brain. Because the retina is the only portion of the central nervous system optically accessible, NVC can also be observed in vivo at cellular resolution noninvasively. Administering a visible flickering light to the eye activates amacrine cells and ganglion cells in the inner retinal layers, triggering the dilation of primary arterioles on the retinal surface, leading to enhanced blood flow throughout arteriolar, capillary, and venular networks within the retina (*14*). As part of the central nervous system, the retina shares similarities with the brain in terms of anatomy and functionality (*15*). Consequently, several major neurological diseases, including Alzheimer’s disease, manifest early in the retina, with retinal NVC dysfunction being one of the earliest signs (*14*–*18*). Therefore, cellular resolution in vivo retinal NVC imaging holds the potential to provide key biomarkers of neurodegenerative disease much earlier in the disease process than conventional neuroimaging methods or clinical examinations (*19*).

Nevertheless, investigating NVC in vivo remains challenging and many of its aspects are still unknown. The dilation of blood vessels in response to light stimulation is minimal, typically only a few micrometers (*20*), necessitating a system with high resolution and contrast for accurate measurement. Furthermore, NVC is a dynamic process that requires continuous monitoring to track temporal changes in vessel diameter. In addition, even without external stimulation, the tone of retinal arterioles is modulated by spontaneous rhythmic fluctuations in blood vessel size due to the cardiac cycle and vasomotion (*21*–*23*). To be able to discriminate vasodilation provoked by flicker stimulation in the NVC process from intrinsic and spontaneous vasomotion and cardiac pulsation, an imaging method with both high spatial and temporal resolution is necessary. A better characterization of these three different sources of vasodilation is crucial not only to gain a deeper understanding of NVC process but also to uncover key biomarkers.

The dynamic vessel analyzer (DVA) is the clinical gold standard method for assessing the effect of light stimulation on retinal vessels using a conventional fundus camera. However, because of its low spatial resolution, DVA cannot directly image the vessel walls and thus presents a low precision measurement (*24*). Optical Coherence Tomography (OCT) technique, a gold standard in retinal imaging, can also be used to measure the diameter of blood vessels at a high resolution in the axial direction. However, OCT inevitably suffer from time-varying speckle noise, which adversely affect the precision of the measurement (*25*, *26*).

By correcting for eye aberrations, adaptive optics (AO) methods can reach transverse resolution in the retina down to 2 μm (*27*, *28*). Owing to this resolution, blood vessel walls can be resolved (*29*–*38*), which makes AO-based ophthalmoscopes ideal to measure the slight widening of vessels when the retina is exposed to light stimulation. Using AO-based ophthalmoscopes, researchers were able to measure the increase in lumen diameter of capillaries induced by flicker stimulation (*39*–*42*). However, measurement of vessel caliber was only done before, during, and after the flicker stimulation, thus not continuously over time. The low number of time-point measurements is due to low frame rate or low image contrast/signal-to-noise ratio, requiring averaging for precise vessel diameter assessment. Moreover, images were acquired in a narrow field of view (FOV), making it challenging to image the same vessel or capillary over several seconds. The constraining number of time-point measurement prevents accurate NVC characterization as spontaneous vasodilation is not taken into account.

**Fig. 1.**
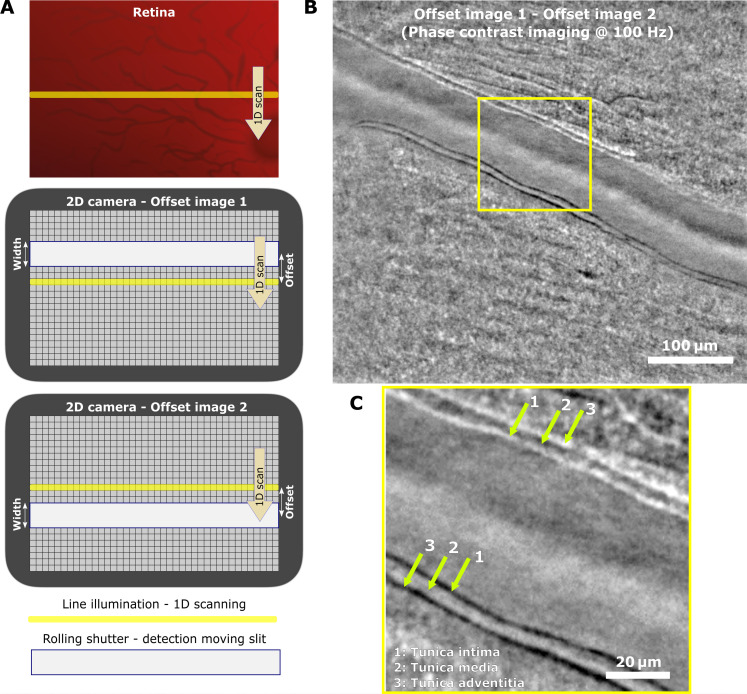
Principle of the AO-RSO method for high-speed and large FOV phase contrast retinal imaging. (**A**) The line illumination is formed using a Powell’s lens, and it is scanned in 1D over the retina using a galvanometer mirror. The rolling shutter detection of a 2D camera is used as a moving slit, synchronized with the line illumination and with a spatial offset to detect phase contrast information. Two consecutive images are acquired with opposite offsets, typically at 200 Hz. (**B**) Phase contrast image is generating by subtracting these consecutive images, after correcting for eye motion, allowing us to achieve a phase contrast image frame rate of 100 Hz over a 4.5° × 2.5° FOV. (**C**) Magnified image showing the vessel wall structure, which became visible owing to the phase contrast.

To address these shortcomings, we developed an imaging method that enables direct visualization of vessel walls of varying calibers in both arteries and veins, with high spatial and temporal resolution. The proposed method is the AO rolling slit ophthalmoscope (AO-RSO), a method using a line scanning illumination synchronized with the rolling shutter of a high-speed scientific complementary metal-oxide-semiconductor (sCMOS) camera for the detection (*43*, *44*). By imposing an offset between the illumination line and the rolling shutter of the camera, phase contrast images, similar to those obtained with the off-axis AO scanning laser ophthalmoscope (AO-SLO) (*31*, *32*, *37*, *38*), can be generated (see Fig. 1). Contrary to the off-axis AO-SLO, given the use of two-dimensional (2D) camera, the AO-RSO can generate phase contrast images at a high frame rate (up to 100 Hz) and large FOV (4.5° × 2.5°). Moreover, because only 1D scan is necessary in the AO-RSO, the effect of motion-induced image distortion is strongly reduced. Using the AO-RSO, we were able to reliably measure flicker-induced vasodilation over time with a spatial precision of 100 nm and a temporal precision of 10 to 100 ms. With such high precision, we characterized the NVC response in terms of vasodilation over eight healthy participants (see sections “Measuring the dynamics of retinal arteries with and without flicker stimulation” and “Study of the vascular diameter response to flicker stimulus on eight healthy participants”). Last, we investigated how flicker-stimulated vasodilation is influenced by arterial diameter (see the section “Influence of arterial diameter on flicker-induced vasodilation”), flicker stimulus duration (see the section “Studying the effect of flicker duration”), and vessel location (see the section “Study of simultaneous flicker response of one vein and two arteries perfusing different areas of the retina”), providing insights into potential biomarkers for vision-impairing conditions and neurodegenerative diseases.

## RESULTS

### Measuring the dynamics of retinal arteries with and without flicker stimulation

To measure the dynamics of retinal arteries in healthy participants, imaging was conducted under two conditions: (i) during 40 s with no stimulation and (ii) during a green flicker test. The flicker test involved 10 s without stimulation, 20 s with 10-Hz flicker stimulation, and another 20 s without stimulation. See Materials and Methods for more information about flicker parameters.

**Fig. 2.**
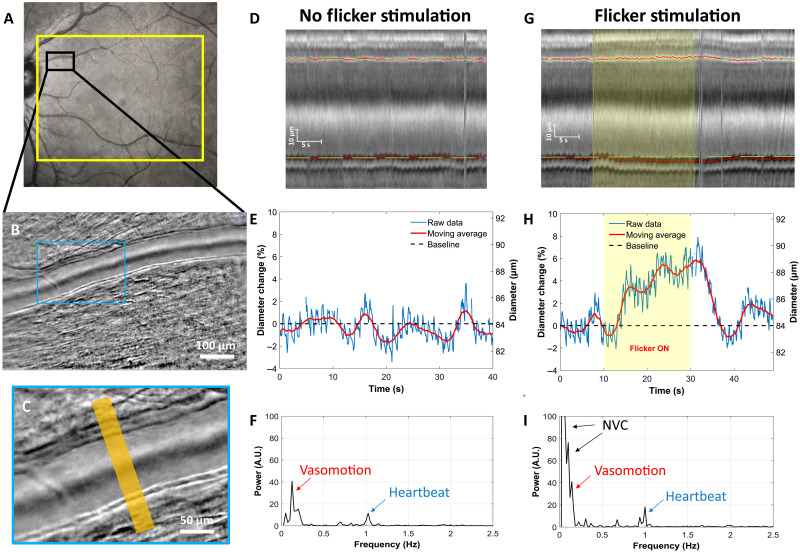
Measuring vascular dynamic at a high resolution. (**A**) Subject’s fundus image acquired with Spectralis (Heidelberg, Germany), where the yellow rectangle indicates the stimulated area during the wide-field flicker test and the black rectangle shows the imaged area. (**B**) AO-RSO phase contrast image with a 2.5° × 4.5° FOV and high-contrasted vessel wall. (**C**) Magnified region of the phase contrast image (blue rectangle), where the orange area indicates the section of the vessel where the lumen diameter is measured. (**D**) Kymograph of the artery without flicker stimulation representing the profile of the vessel over time; green dashed lines represent the baseline position of the vessel wall. (**E**) Artery’s lumen diameter over time without flicker stimulation. (**F**) Power spectral density of the lumen diameter data. A.U., arbitrary units. (**G**) Kymograph of the artery with flicker stimulation; the stimulation is represented as a yellow rectangle. (**H**) Artery’s lumen diameter over time with flicker stimulation. (**I**) Power spectral density of the lumen diameter data during flicker stimulation. Note in (E) and (H) that both relative diameter change (left axis) and diameter change (right axis) are presented.

Figure 2 presents the vascular response to flicker stimulus for one subject measured using the phase contrast AO-RSO method. A flicker stimulation of a 25° × 20° field was done centered on the fovea (Fig. 2A). The AO-RSO system was used to image an artery perfusing the fovea at 100 Hz (Fig. 2B). From the recorded images, a section of the vessel was selected (Fig. 2C), and kymographs were generated (Fig. 2, D and G), enabling us to quantify the evolution of the vessel’s lumen diameter over time (Fig. 2, E and H). The baseline diameter is defined as the average diameter of the vessel during the first 10 s (without flicker). Without stimulation (Fig. 2E), the vessel’s diameter is modulated by two components, which is even more evident when analyzing the power spectral density (Fig. 2F): (i) cardiac cycle, with a rhythmic dilation of around 1 Hz (in blue); and (ii) spontaneous vasomotion, with a frequency of around 0.1 Hz (in red), due to alternating smooth muscle dilation and constriction (*21*–*23*).

Figure 2 (G and H) shows the evolution of the diameter of an artery during a flicker test. When the stimulation starts (Fig. 2H at *t* = 10 s), the diameter of the artery increases rapidly at around 1%/s for ~5 s. Then, the artery continues to dilate steadily at 0.1 to 0.2%/s until a maximum dilation is reached around the end of the 20-s flicker stimulation. After the end of the stimulation, the artery contracts back to its diameter pre-flicker. On some acquisition, this contraction undershoots under the baseline diameter before dilating back to the baseline diameter (see figs. S1 to S8 in the Supplementary Materials). The power spectrum density (Fig. 2I) reveals an additional low-frequency content (<0.1 Hz) with flicker stimulation, indicating NVC activity. This component shows a higher magnitude compared to baseline conditions without flicker. The general trend of the vessel’s diameter evolution during a 20-s flicker test is consistent with what has been described with fundus camera imaging using the DVA (*45*–*47*). However, owing to our high spatial resolution and high-contrasted vessel wall visualization, our approach can precisely distinguish, in space/time and frequency domain, the contribution of heartbeat, vasomotion, and NVC during the full time course with high precision and on a single image acquisition.

### Study of the vascular diameter response to flicker stimulus on eight healthy participants

**Fig. 3.**
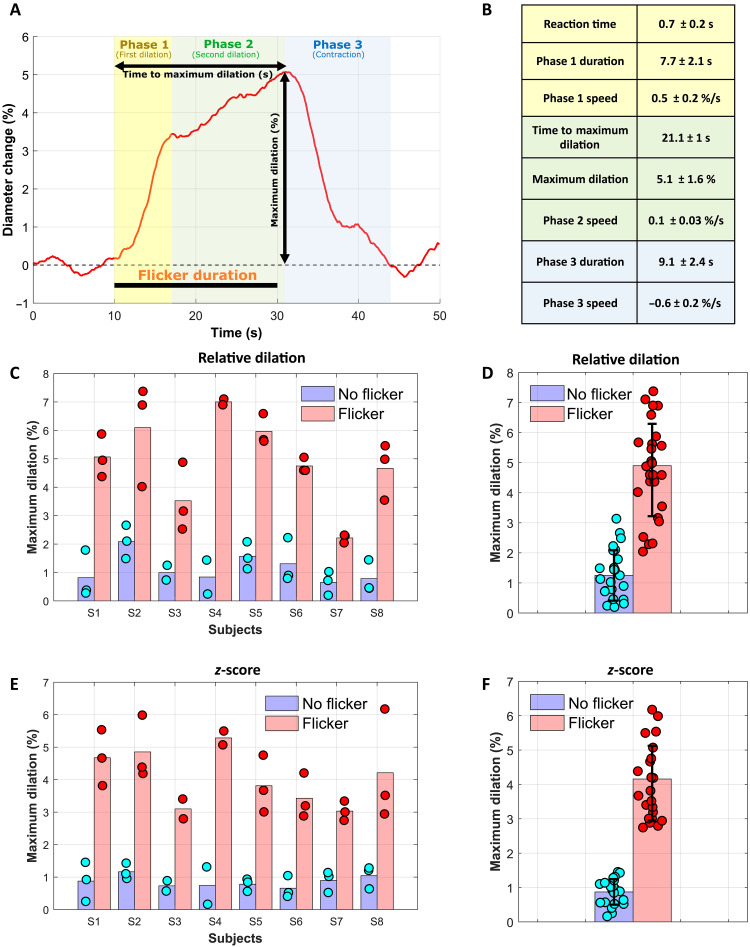
Characterization of NVC over the population. (**A**) Averaged vascular response to flicker stimulation over the population. Various temporal aspects of the flicker response, represented on this graph, were measured for each acquisition across eight subjects. (**B**) Average and SD over the population of these parameters across all acquisitions. (**C**) Average (bar) and individual (dots) maximum relative (%) dilation for each subject without flicker stimulation (blue) and with flicker stimulation (red). (**D**) Average and SD of the relative maximum dilation for all acquired data without flicker (blue) and with flicker (red) stimulation. (**E** and **F**) Average and individual measurement for each subject and for all acquired data of the *z*-score, respectively.

The same protocol was conducted on eight healthy participants. For each subject, three acquisitions without stimulation and three acquisitions with a flicker stimulation were taken. When the retina is exposed to a flicker stimulus, a triphasic behavior can be observed (Fig. 3A). The average and SD of the flicker-induced vascular response over the population can be found in Fig. 3B.

Following flicker stimulus (phase 1), the artery starts to dilate with a reaction time of 0.7 ± 0.2 s, with an average speed of 0.5 ± 0.2%/s until reaching a first maximum at 7.7 ± 2.1 s after the onset of stimulation. Then, the artery continues to dilate at a lower rate of 0.1 ± 0.03%/s (phase 2) until reaching the maximum dilation of 5.1 ± 1.6% after 21.1 ± 1 s of the stimulation start. Note that the maximum dilation was reached after the end of the stimulus (around 1.1 ± 1 s after), which is consistent with a previous work from Riva *et al.* (*20*). After the end of the stimulation (phase 3), the artery starts to contract back into its original diameter at a speed of −0.6 ± 0.2%/s, until reaching the baseline value at 9.1 ± 2.4 s.

Figure 3C presents the relative maximum dilation on all acquisitions for each subject. For all the subjects, the maximum dilation was significantly higher with flicker (red bars) than without (blue bars, *P* < 0.0001). Without flickering, we measured an averaged maximum dilation of 1.15 ± 0.7%, which is due to vasomotion. From our study, we have seen large differences between subjects for the amplitude of vasomotion, varying from 0.2 to 2.7%. Because of this high variability, when considering each acquisition individually, one can still see some slight overlap (*48*) (Fig. 3D). To minimize the effect of vasomotion, we normalized each measurement to the SD of the baseline (named *z*-score) (*48*), as it is shown in Fig. 3 (E and F). At this time, a clear border can be set between the flicker-induced vessel response and spontaneous vasomotion, revealing a promising biomarker for NVC (Fig. 3F). The absolute maximum dilation in micrometers for each subject with and without flickering is shown in fig. S9 in the Supplementary Materials, highlighting our capacity to measure micrometer-scale changes of vessel diameter.

### Influence of arterial diameter on flicker-induced vasodilation

**Fig. 4.**
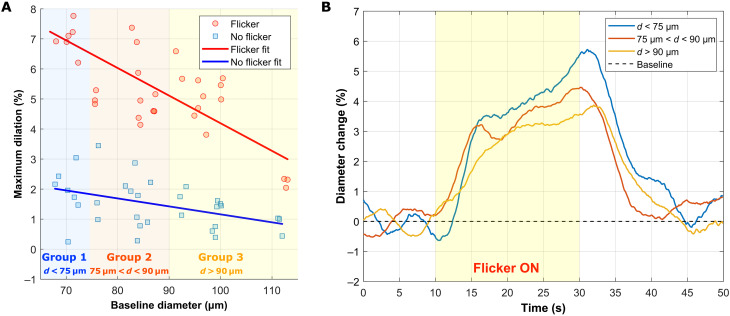
Influence of arterial diameter on NVC response. (**A**) Maximum dilation for each flicker stimulation (red) and no flicker acquisition (blue) as a function of baseline vessel diameter. (**B**) Averaged graph of every flicker test grouped by diameter (*d*): blue: <75 μm; red: between 75 and 90 μm; yellow: >90 μm.

We investigated the influence of the diameter of the artery on the response to flicker stimulation. In Fig. 4A, the maximum dilation is represented as a function of baseline arterial diameter in the cases of flicker and nonflicker (30 data for each case, collected over eight subjects). Whereas in the nonflicker case, no correlation can be cleared noted (R^2^ = 0.19), the flicker case shows a linear correlation (R^2^ = 0.19, *P* < 0.0001), where the smaller the artery, the larger the dilation (slope = −0.09%/μm). To get more insight into the temporal aspect of the dilation depending on the size of the arteries, we separated the subjects into three groups depending on the baseline diameter (*d*) of the imaged arteries: *d* < 75 μm (group 1), 75 μm < *d* < 90 μm (group 2), and *d* > 90 μm (group 3). In Fig. 4B, the average curves from each group are plotted. Notably, the maximum dilation is most pronounced in the *d* < 75 μm group and least pronounced in the *d* > 90 μm group. However, it is important to note that the absolute maximum dilation is about the same for all three groups (around 3.7 μm; see fig. S10 in the Supplementary Materials). Moreover, directly after the onset of flicker, in phase 1, the three groups exhibit a drastically different dilation speed.

### Studying the effect of flicker duration

**Fig. 5.**
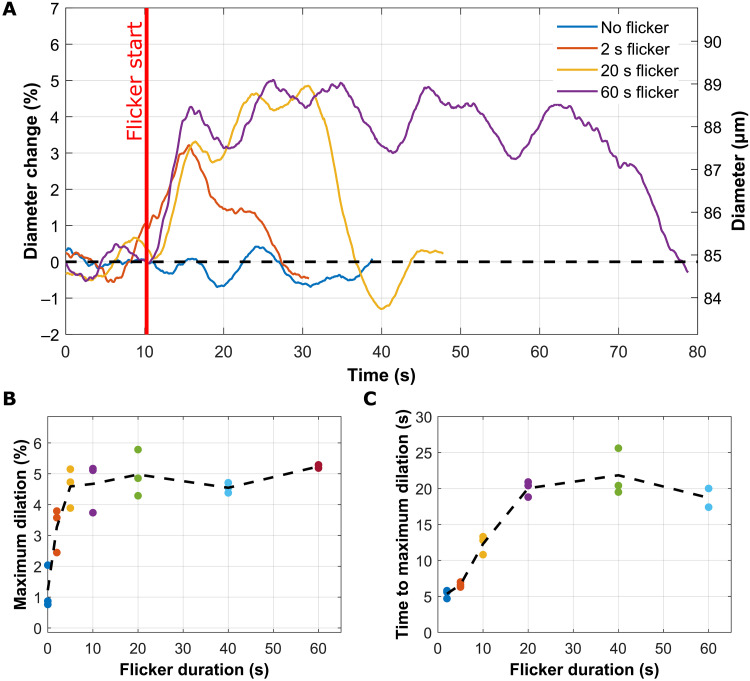
Effect of flicker duration on NVC response. (**A**) Moving average diameter over time for different flicker durations, with flicker stimulation starting at *t* = 10 s. For clarity, only flicker durations of 0, 2, 20, and 60 s are represented (see fig. S11 in the Supplementary Materials for the other cases). Note that both relative diameter change (left axis) and diameter change (right axis) are presented. (**B**) Averaged time to maximum dilation over flicker duration. (**C**) Averaged maximum dilation over flicker duration.

To study how the flicker duration may influence the vascular response, we conducted a flicker test of increasingly longer stimulation times on a single subject (0, 2, 5, 10, 20, 40, and 60 s). Three acquisitions were taken for each flicker stimulation duration and then averaged. The results from 2, 20, and 60 s are presented in Fig. 5A (see fig. S11 in the Supplementary Materials for the results of each flicker duration).

For 2-s flicker duration, although a clear increase in the vessel’s diameter can be observed compared to without stimulation, the dilation is less pronounced and with a different shape (biphasic behavior) compared to the triphasic behavior of the standard 20-s flicker stimulation. As shown in Fig. 5A, an ~3% maximum dilation for 2-s stimulation was obtained. The maximum dilation is reached around 6 s after the beginning of the flicker stimulation, therefore 4 s after the end of the flicker. This delayed dilation is probably linked to the first dilation observed for 20-s flicker duration (phase 1) described in Fig. 3A. Such a phenomenon may indicate that the first dilation (phase 1) and the second dilation (phase 2) have different origins. The phase 1 dilation seems to be independent of the stimulation duration, with a peak around 6 to 7 s after the beginning of the stimulation (also observed in the case of 5-s duration; see fig. S11 in the Supplementary Materials). On the other hand, the phase 2 slower dilation only happens if the duration is longer (already present for 10-s duration; see fig. S11 in the Supplementary Materials). In phase 2, the time to maximum dilation seems to increase linearly until reaching a plateau around 20 s, for flicker durations of 20, 40, and 60 s (Fig. 5B). For some of these long flicker durations, the vessel’s diameter starts to slightly decrease even before the end of the stimulation as it can be observed for 60 s in Fig. 5A. Last, Fig. 5C shows the maximum dilation over flicker duration, where a fast increase is noticed for short durations (below 5 s), going from 0.41 to 4.2% followed by a saturation around 20 s for 5% dilation.

### Study of simultaneous flicker response of one vein and two arteries perfusing different areas of the retina

**Fig. 6.**
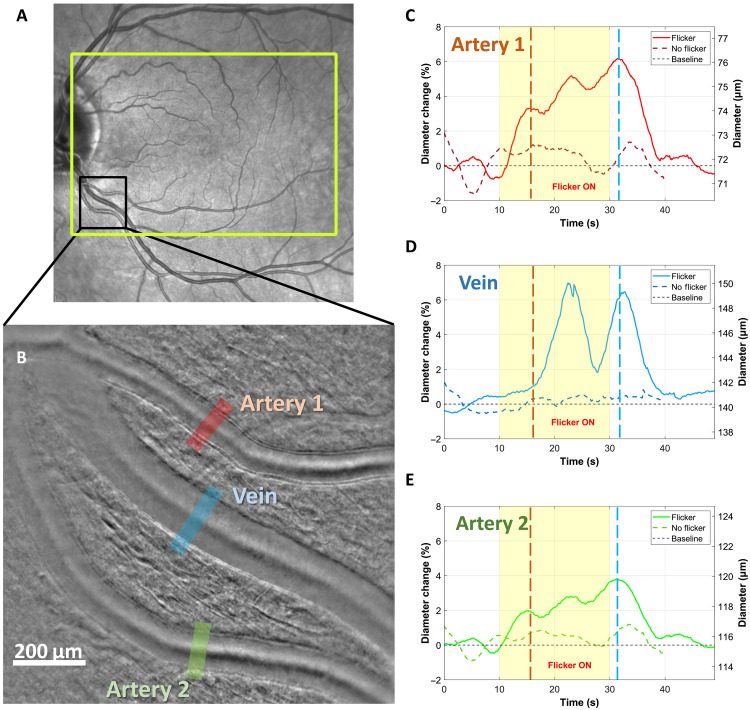
Influence of vessel localization on NVC response. (**A**) Subject’s fundus image acquired with Spectralis (Heidelberg, Germany), where the yellow rectangle indicates the stimulated area during the wide-field flicker test and the black rectangle shows the imaged area. (**B**) AO-RSO phase contrast image, with an FOV of 4.5° × 4.5°, where we can identify two arteries and a vein. The area where the lumen diameter was measured is represented with red, blue, and green rectangles. (**C** to **E**) Average of two trials of the evolution in time of the vessel diameter for artery 1, vein, and artery 2, respectively, with and without flicker. Note that both relative diameter change (left axis) and diameter change (right axis) are presented.

We took advantage of our system’s large FOV to measure the effect of flicker stimulation on three vessels simultaneously. On one subject, we selected an area of interest where two arteries and one vein could be imaged within a 4.5° × 4.5° FOV (Fig. 6, A and B). In addition, the two arteries supply blood to distinct regions: artery 1 directly supplies the fovea, whereas artery 2 supplies the peripheral retina, which comprised nonstimulated areas. The diameter of all three vessels was measured and their evolution over time with and without flicker is represented in Fig. 6 (C to E).

Figure 6 (C and E) shows that the temporal evolution of both arteries, with and without flicker, is mostly synchronized and similar. This resemblance may come from their shared origin as daughter branches of the same artery. However, artery 1, which perfuses directly the fovea (stimulated zone), exhibits a larger dilation of 6.2% than artery 3 (supplying mostly a nonstimulated peripheral area) with a maximum dilation of 3.8%. On the other hand, the temporal evolution of the vein’s diameter differs considerably from that of the two arteries (Fig. 6D) in both flicker and nonflicker cases. For the nonflicker case, the vasomotion seems decorrelated and with a smaller magnitude compared to the arteries. For the flicker case, the vasodilation response to flicker stimulus presents a unique behavior, without a clear triphasic pattern. First, the vein reaction time is around 1.3 s compared to 0.7 s from arteries, with a more gradual response (0.12%/s) compared to the arterial fast dilation in phase 1. This slow vein diameter increase coincides with the time of phase 1 (Fig. 3B). Second, in phase 2, 6.7 s after the stimulus onset, the vein diameter increases rapidly and the vasomotion amplitude seems much larger than for arteries, thus reaching a maximum dilation superior from those of the arteries (7%). Moreover, vasomotion seems unexpectedly in phase with a slight delay compared to the arteries. Last, in phase 3, after the stimulus, the reaction time before the decrease in diameter is around 2.6 s, therefore delayed compared to the arteries (1.1 s).

## DISCUSSION

We developed a noninvasive, high-resolution, high–frame rate, large FOV phase contrast imaging system to characterize, with unprecedented spatiotemporal precision, the effect of flicker light stimulation on large arteries and veins of healthy participants. The proposed method enabled reliable measurement of flicker-induced vasodilation with a spatial precision of ~100 nm (see Materials and Methods) and a temporal resolution of 10 to 100 ms. In addition, the large FOV imaging and minimized effect of motion-induced distortion provided by the system ensure high robustness, enabling continuous data acquisition for up to several minutes (see fig. S12 in the Supplementary Materials), with data loss occurring exclusively during subjects’ blinks and microsaccades.

The complete temporal dynamics of retinal artery diameters were measured in eight healthy participants, both with and without flicker stimulation (Figs. 2 and 3 and figs. S1 to S8 in the Supplementary Materials). The continuous high-speed measurements provided by our system allowed simultaneous observation of the effects of the cardiac cycle, vasomotion, and flicker stimulation on the vessel diameter. These results emphasize how crucial it is to capture the full temporal profile of dilation to study NVC effectively, rather than relying solely on pre- and poststimulation diameter measurements, as it is commonly done in retinal neurovascular studies. With flicker stimulation, we showed that the vascular response has a triphasic behavior (Fig. 3), with a significant maximum dilation (5.2 ± 1.6%) compared to the nonflicker case (1.15 ± 0.7%, *P* < 0.0001). Figure 3 also showed a variability of maximum dilatation with flicker among subjects (varying from 2 to 7.4%) as well as a variability among different acquisitions for a single subject. The intrasubject variability can be attributed to the occurrence of vasomotion, with large amplitude variability and its phase, affecting the dynamic of the diameter change (*48*). In addition, the intersubject variability could be explained by the dependence on the artery caliber. We have shown that smaller arteries have a more significant vessel dilation in response to flicker stimulus (Fig. 4, *R*^2^ = 0.58, *P* < 0.0001), which is in accordance with a previous study (*49*). Another factor that may influence the intersubject variability is the spatial selectivity of NVC in the retina (*40*). As shown in Fig. 6, the composition (containing more or less activated neurons) of the region, which is perfused by the artery, may also influence the vasodilation response.

The effect of flicker stimulation duration was also evaluated in one subject (Fig. 5). For short stimulation durations (<20 s), a clear increase in vessel’s diameter was noted, although the response was less pronounced compared to the standard 20-s stimulation. Conversely, for stimulation durations longer than 20 s, no further dilation was observed beyond the maximum achieved with 20 s of stimulation. A biphasic behavior was noted for 2- and 5-s flicker duration compared to triphasic for stimulus duration greater equal to 10 s, which may indicate a different origin for phase 1 and phase 2 vascular responses.

Our study indicates that 20-s flicker duration is optimal to achieve maximum vasodilation. We also highlighted that very brief stimulations of just a few seconds can produce substantial vasodilation compared to the baseline. A short flicker test to assess the NVC function could benefit clinical examinations as it could be more comfortable for the patient and allow for a faster examination and image processing.

Although additional acquisitions on multiple subjects would be required to establish a precise characterization of flicker-induced vasodilation on different populations and clinical cases, the presented method and findings pave the way for further exploration of NVC at submicrometer and millisecond resolution. A promising path is the validation of the *z*-score as a reliable biomarker to detect health NVC response. As we have shown in Fig. 3 (E and F), the *z*-score allows to set a clear boarder between nonflicker and flicker-induced vasodilation. A precise biomarker and characterization, in space and time, of the mechanisms underlying physiological and/or pathophysiological NVC responses can facilitate current queries in neurovascular metabolism research, could assist the evaluation of retinal and neurodegenerative diseases, and may ultimately lead to the development of therapies to prevent the breakdown of NVC.

Another promising avenue lies in our method’s unique capability to distinguish vasomotion from light-induced vasodilation (the latter resulting from NVC). Vasomotion plays a crucial role in maintaining vascular health and enhancing local perfusion, and it is known to be affected by conditions such as diabetes and hypertension. The ability to monitor vasomotion—either independently or alongside light-induced vasodilation—could support the identification of biomarkers for assessing vascular function. Moreover, the interaction between vasomotion and NVC remains poorly understood. Our method may help clarify how these processes interact and how their relationship is affected in vascular and neurovascular dysfunction.

## MATERIALS AND METHODS

### Subjects

Eight subjects, ranging in age from 24 to 33 years (S1 = 24, S2 = 25, S3 = 26, S4 = 25, S5 = 27, S6 = 27, S7 = 28, and S8 = 33 years old) and free of ocular disease, participated in the experiments. All subjects had a spherical equivalent refraction between 0 and −4.5 diopters. All had normal intraocular pressure (IOP) and an appearance of optic disk and fundus. Patients were included in an institutional study carried out according to the principles outlined in the Declaration of Helsinki and approved by the ethics committee (Comité de Protection des Personnes; registered in ClinicaTrial.Gov NCT04128150). Written informed consent was obtained from all participants following an explanation of experimental procedures and risks both verbally and in writing. The total irradiance for the imaging and AO light sources was 1700 and 3.8 μW, respectively, which is below the ocular safety limits stipulated by the International Organization for Standardization (ISO) standards for group 1 devices.

### Delivery of flicker stimulation

A stimulation screen (Waveshare, China) was integrated into the imaging system. The screen provided a 25° × 20° illumination region centered on the fovea. The screen was combined with the imaging system using a pellicle beam splitter. A programmable, stable fixation target was provided for the subject during all stimulus conditions. The flicker stimulation was generated and delivered using custom Python software, allowing the simultaneous start of the recording with the imaging system and the beginning of the flicker test protocol. The full-field flicker stimulus used in this experiment was green (530 nm, 40-nm full width at half maximum) with a square-wave modulation of the entire field at 50% duty cycle with a maximum illuminance of 7.3 lux and a minimum illuminance of 0 lux. These illuminance intensities and modulation rates were chosen to be similar to the previous literature studying NVC in the retina to induce high metabolic activity (*20*, *50*, *51*).

### Imaging system

We used our AO-RSO, which has been previously described elsewhere (*43*, *44*). In short, it consists of a 2D camera–based, nonconfocal, split detection AO-line scanning ophthalmoscope. The light emitted from a SLED (Thorlabs) is projected in the retina into a line pattern of 10-μm width using a Powell lens. The light backscattered by the retina is then detected using the rolling shutter of the detection camera (ORCA-Fusion, Hamamatsu). The scans performed by the rolling shutter and the galvanometer mirror of the illumination are synchronized via custom Matlab (MathWorks, Natick, MA) software. To achieve phase contrast imaging of the vessel walls, a 10-μm offset between the illumination laser line and the exposed pixels of the rolling shutter is imposed. The exposure time of each line of pixel of the rolling shutter is chosen at 300 μs, thus giving an effective width of the rolling shutter of 50 μm in retinal space. Consecutive positive and negative offset images are alternated and subtracted to achieve split detection imaging, further increasing the phase contrast of images. A similar method has been previously described, but it did not use a Powell lens to generate line illumination or AO to achieve micrometer-scale resolution in the retina and resolve vessel walls (*52*). The AO-RSO allows us to acquire images at a high resolution (2 μm) and high speed (100 Hz), using phase contrast imaging on a large FOV (2.5° × 4.5°) and without apparent motion-induced image distortion. Last, owing to the vertical scanning, one can increase the FOV by sacrificing the frame rate (e.g., 4.5° × 4.5° for 50 phase contrast images/s) or a narrower FOV for a faster acquisition rate.

### Image acquisition protocol

All imaging sequences were collected with the room lights off. The subject’s eye was cyclopleged and dilated using 0.5% tropicamide. The eye and head were aligned with the imaging system using a chinrest mounted to a motorized *XYZ* translation stage. Correct imaging focus was realized by optimizing vessel wall contrast using the real-time displayed images. For each subject, an artery upstream of the fovea in the nasal region was selected using a fundus camera image of the subject. The imaging region was chosen along the selected artery and at ~8° eccentricity. Once the imaging region was selected, three acquisitions of 40 s without stimulation were taken. Then, three flicker tests, each consisting of 10 s of recording without stimulation, 20 s of flicker stimulation, and 20 s without stimulation, were taken. Between each flicker test, a 1-min break was included, which is large enough given that the time to reach the baseline after the flicker stimulus is around 20 s. To further ensure that 1-min break is enough and do not influence any cumulative effect, we conducted consecutive flickers with 30-s pause between them during 3 min, with no cumulative effect visible (see fig. S12 in the Supplementary Materials). Recording by the imaging system was synchronized with the stimulation channel. The system acquired images continuously during the entirety of each 40-s baseline recording and during each 50-s flicker tests. Images are sequentially acquired with opposite offsets, typically at 200 Hz. Then, a pair of sequential acquired images, with opposite offsets, are subtracted to generate phase contrast images. Thus, the system provides phase contrast imaging at 100 Hz on a 2.5° × 4.5° FOV, allowing visualization of 1.2-mm sections of horizontal blood vessels and making it possible to keep the vessel of interest inside the imaging FOV despite imperfect eye fixation, which is unavoidable during 50-s acquisitions.

### Image processing

Images were first registered using a custom Matlab (MathWorks, Natick, MA) phase correlation algorithm (*53*). Images were averaged in groups of 10 to enhance the signal-to-noise ratio, thereby producing a sharp image of the blood vessels every 100 ms. This approach was used to enhance the signal-to-noise ratio while maintaining the capability to monitor blood vessel’s fluctuation in diameter due to the cardiac cycle in all subjects. Image sequences with too much movement or blink occurrences were detected and discarded.

### Vessel wall detection and measurement

From the registered and averaged images, a 30-μm segment of the vessel was chosen. For each averaged image, pixel lines perpendicular to the vessel’s direction within the selected segment were averaged. These averaged lines, each representing the cross-sectional average of the vessel in the 30-μm segment, were plotted over time to produce a kymograph of the vessel (Fig. 1, C and E). From the kymographs, the position of the vessel’s inner and outer wall was measured on each side. A MATLAB peak detection algorithm was used to determine their positions by identifying the two maxima and two minima in each pixel line of the M-scan corresponding to the tunica intima and the tunica externa of both sides of the vessel. The lumen diameter was defined as the distance between the two inner walls of the vessel. A parabolic fit was implemented to achieve a subpixel measurement of the lumen diameter with a precision of 0.1 pixel size (0.073 μm in our case). To represent the measured diameter, both the raw measurements taken every 0.1 s and the moving average were represented. The moving average was chosen on a 3-s window to filter out the effect of the cardiac cycle. For the measurement of the maximum dilation (represented in Fig. 2A), the moving average was chosen instead of the raw measurement; this was done to filter out the effect of the cardiac cycle on the lumen diameter, where the variations do not reflect the effect of NVC on the vessel.

## Supplementary Materials

### The PDF file includes:

Supplementary Text Figs. S1 to S12 Legends for datasets S1 to S4

### Other Supplementary Material for this manuscript includes the following:

Datasets S1 to S4
