# Supplementary material for: Revealing neurovascular coupling at a high spatial and temporal resolution in the living human retina
Source: Sci Adv. 2025 Jun 27;11(26):eadx2941. doi: 10.1126/sciadv.adx2941 (PMC12204158; doi:10.1126/sciadv.adx2941)
Supplement: Supplementary file 1 — Supplementary Text Figs. S1 to S12 Legends for datasets S1 to S4 [file sciadv.adx2941_sm.pdf]

Science Advances
AAAS

Supplementary Materials for

Revealing neurovascular coupling at a high spatial and temporal resolution in the living human retina

Pierre Senée *et al.*

Corresponding author: Pedro Mecê, pedro.mece@espci.fr; Serge Meimon, serge.meimon@onera.fr

Sci. Adv. **11**, eadx2941 (2025)
DOI: 10.1126/sciadv.adx2941

The PDF file includes:

Supplementary Text
Figs. S1 to S12
Legends for datasets S1 to S4

Other Supplementary Material for this manuscript includes the following:

Datasets S1 to S4

Supplementary Text

Vasodilation over time for all eight subjects

Here, we present one example of acquisition with and without flicker stimulation for each of the eight healthy subjects imaged in this study (S1-S8). In all presented supplementary figures (Fig S1 to FigS8), the reader will find their fundus photography image (A), where the yellow rectangle represents the stimulated area during the wide field flicker test and the black rectangle represents the imaged area, which is highlighted below as a magnified image (B). The profile of the vessel over time without flicker stimulation can be observed in (C), where the dashed white lines represent the baseline position of the wall (tunica intima) of the vessel. (D) shows the artery's diameter over time without flicker stimulation. The vessel profile and vessel diameter over time during flicker stimulation can be seen in (E) and (F) respectively.

Micrometric vascular diameter change in response to flicker stimulus on 8 healthy subjects.

The absolute maximum dilation in micrometers for each subject with and without flickering is shown in Fig. S9.

Influence of Arterial Diameter on Flicker Induced Vasodilation.

Here, we show that when analyzing the data using the absolute diameter change, no correlation between vessel diameter and maximum dilation can be noted (Fig.S10).

Effect of flicker duration on arterial response

Individual vascular responses for different flicker durations are described in Fig. S11. We show that only the first phase is present for durations 2s and 5s, with a maximum dilation achieved after the end of the flicker, around 7s after the beginning of flicker. Phase 2 starts to become apparent for flicker durations of 10s, 20s, 40s and 60s. A saturation of vessel diameter is achieved after around 21s from the beginning of the flicker. For 40s and 60s flicker duration, it is possible to see a very slow decrease of vessel diameter after reaching its maximum.

Consecutive flicker tests and accumulative effect

In order to study how long should we wait before a new NVC imaging, we measured the vessel diameter over a long time with consecutive flicker tests of 20s ON, 30s OFF. Fig S12-A show the results for 2min acquisition without flicker, where vasomotion can be clearly seen. Fig. S12 B,C and D presents long measurement during several flicker tests, highlighting that no accumulative effect is present and that 30s pause (without flicker) after a flicker test, is enough to avoid interference of two consecutive flicker responses. Note in Fig S12 D that a 20s pause is too short, thus contributing to an accumulative effect of the vascular response.

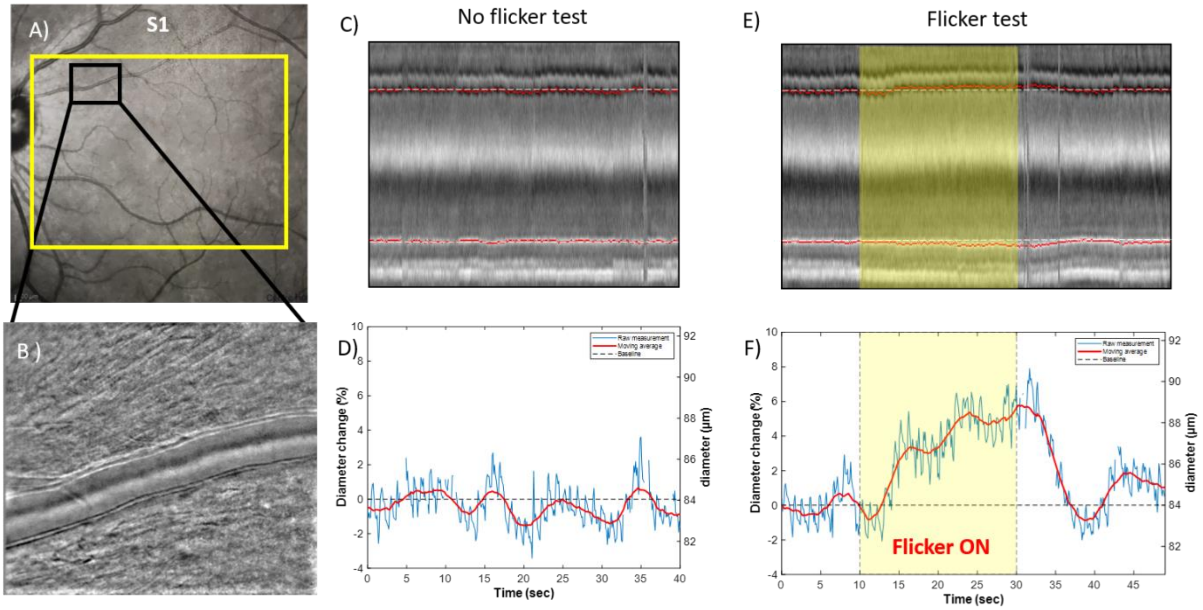

Figure S1: Vascular dynamics without and with flicker for Subject 1 (S1).

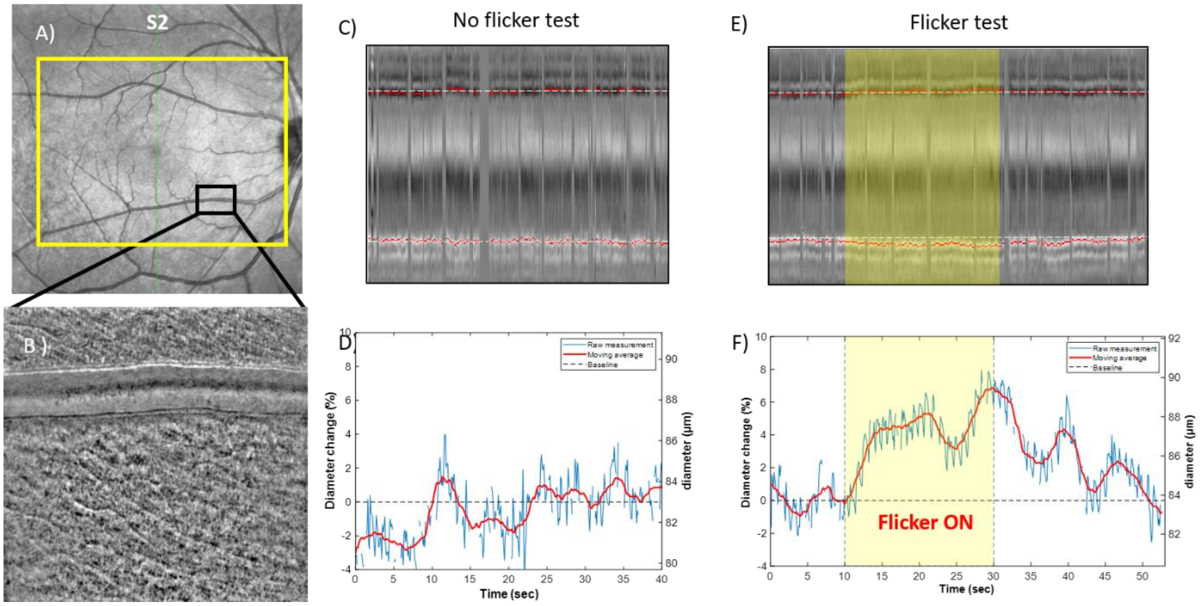

Figure S2: Vascular dynamics without and with flicker for Subject 2 (S2).

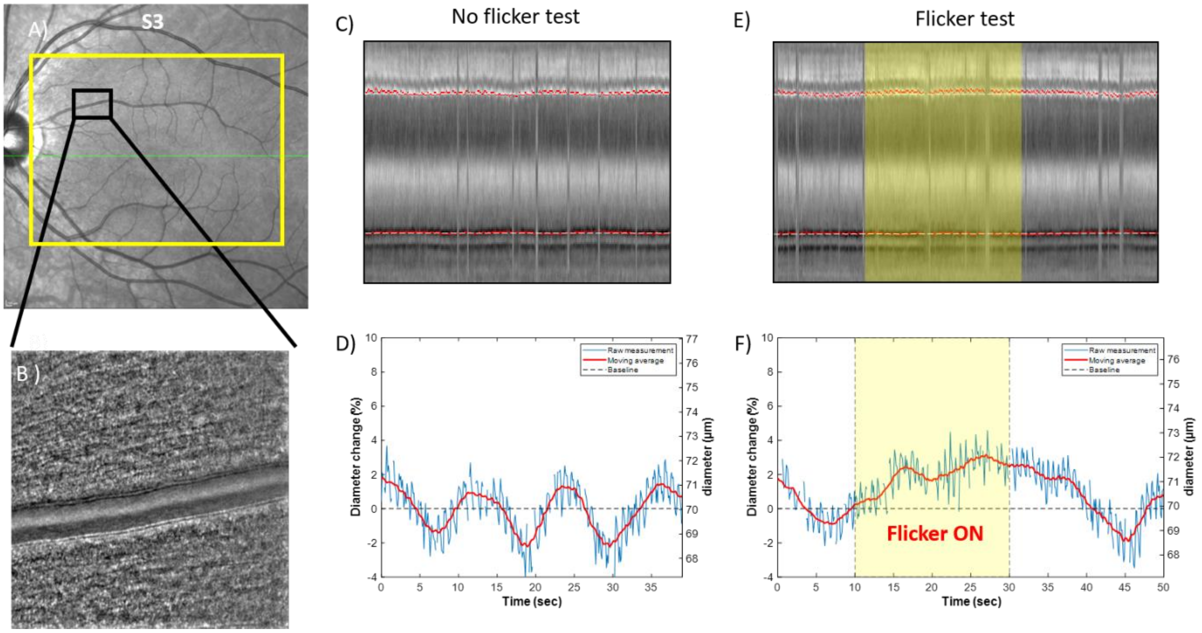

Figure S3: Vascular dynamics without and with flicker for Subject 3 (S3).

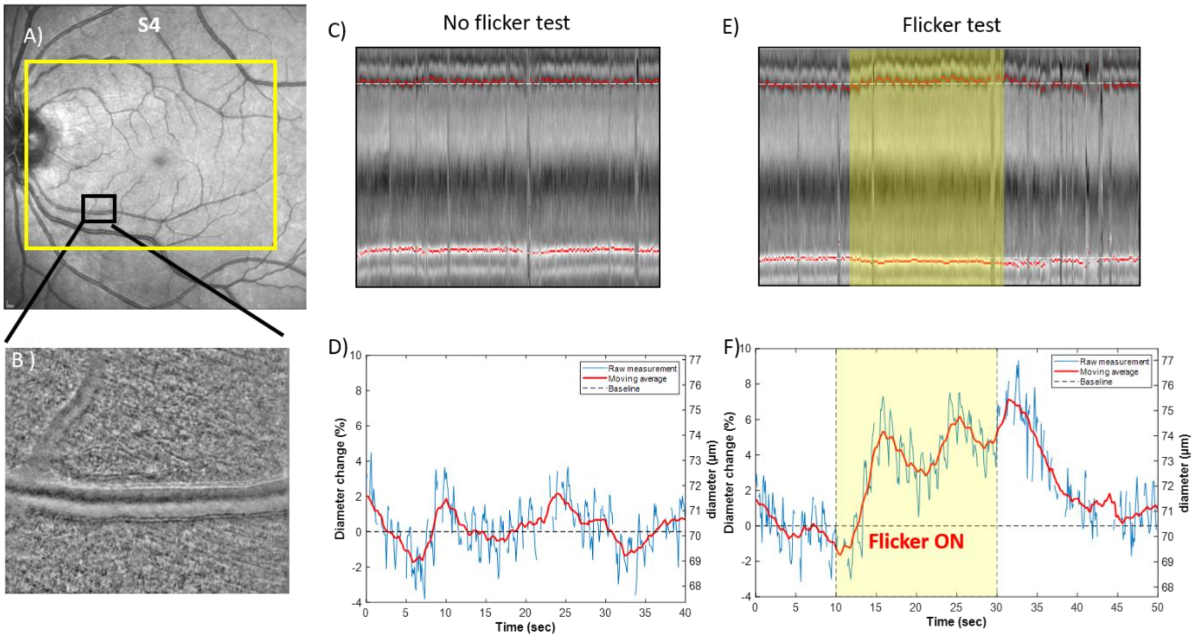

Figure S4: Vascular dynamics without and with flicker for Subject 4 (S4).

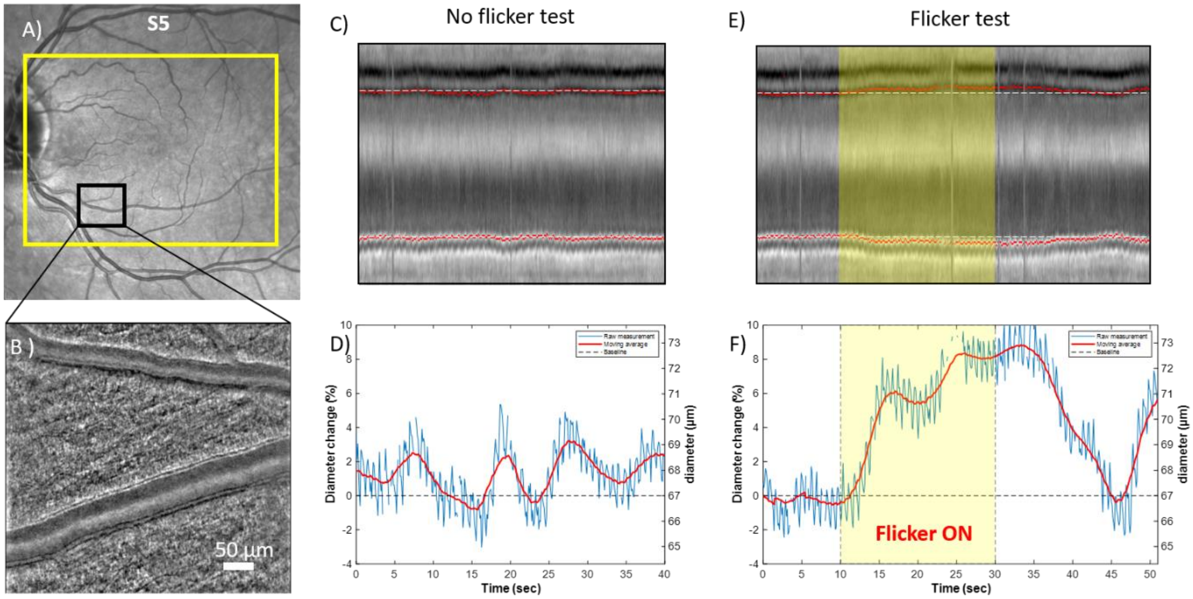

Figure S5: Vascular dynamics without and with flicker for Subject 5 (S5).

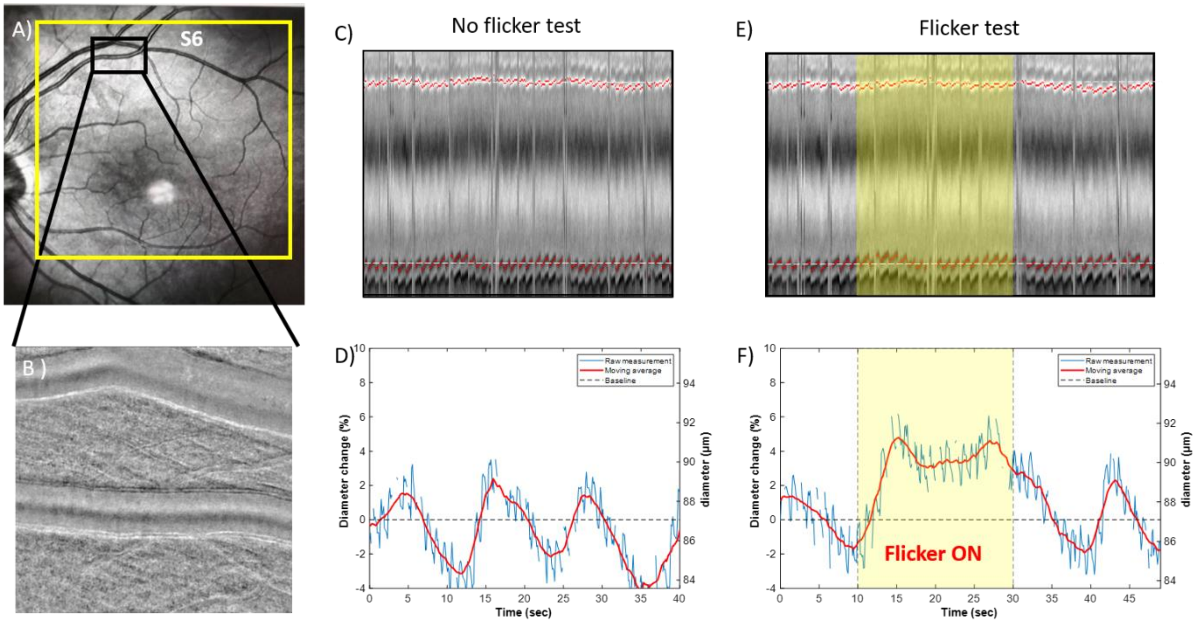

Figure S6: Vascular dynamics without and with flicker for Subject 6 (S6).

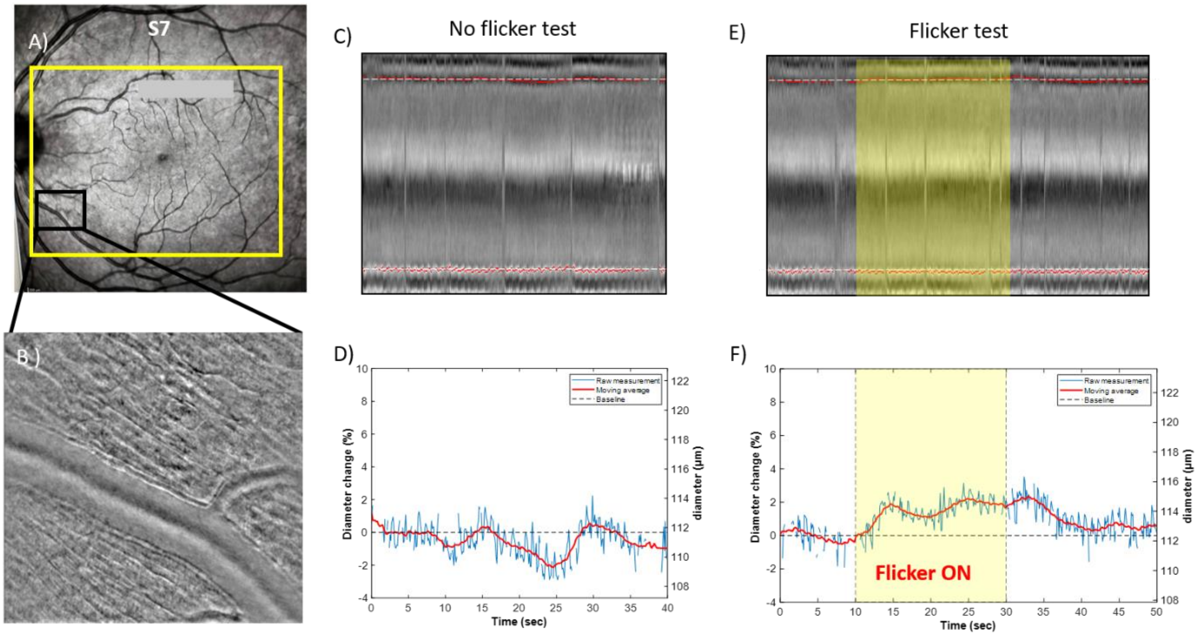

Figure S7: Vascular dynamics without and with flicker for Subject 7 (S7).

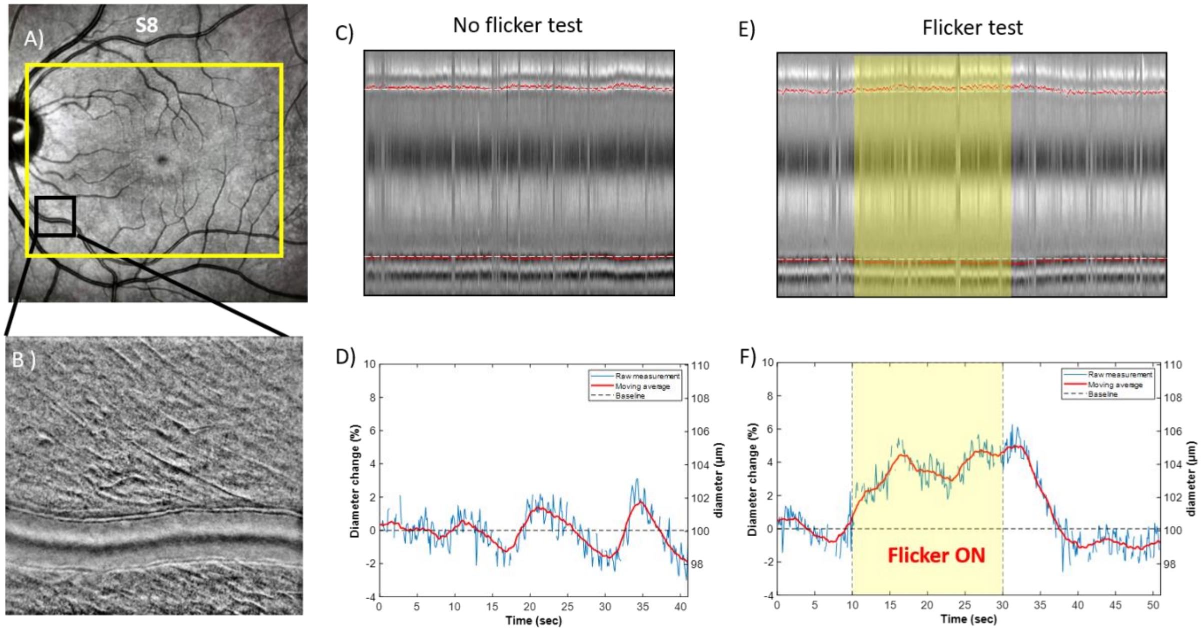

Figure S8: Vascular dynamics without and with flicker for Subject 8 (S8).

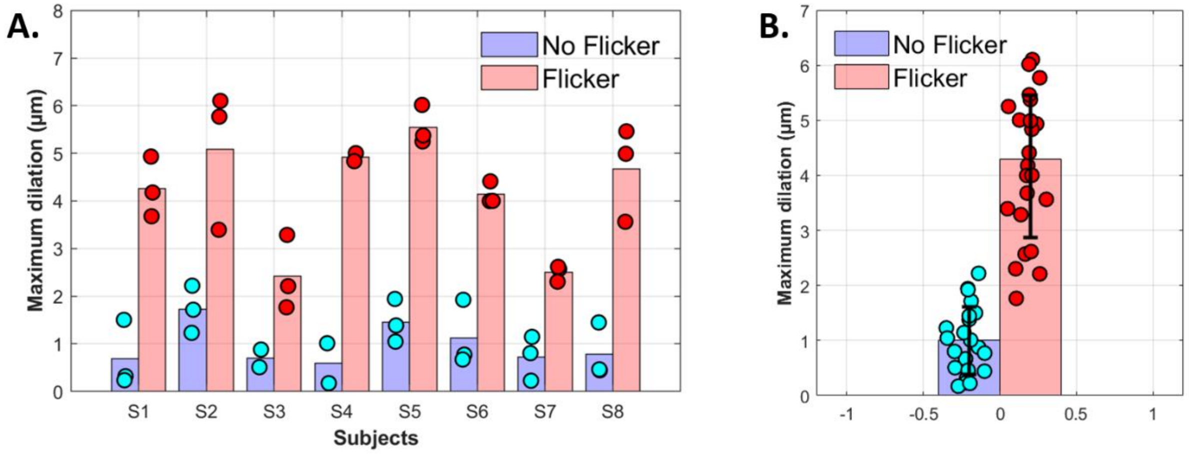

Figure S9: Absolute maximum dilation over the population. A) and B) Average (bar) and individual (dots) measurement of absolute vessel diameter change for each subject and for all acquired data with (red) and without (blue) light stimulus.

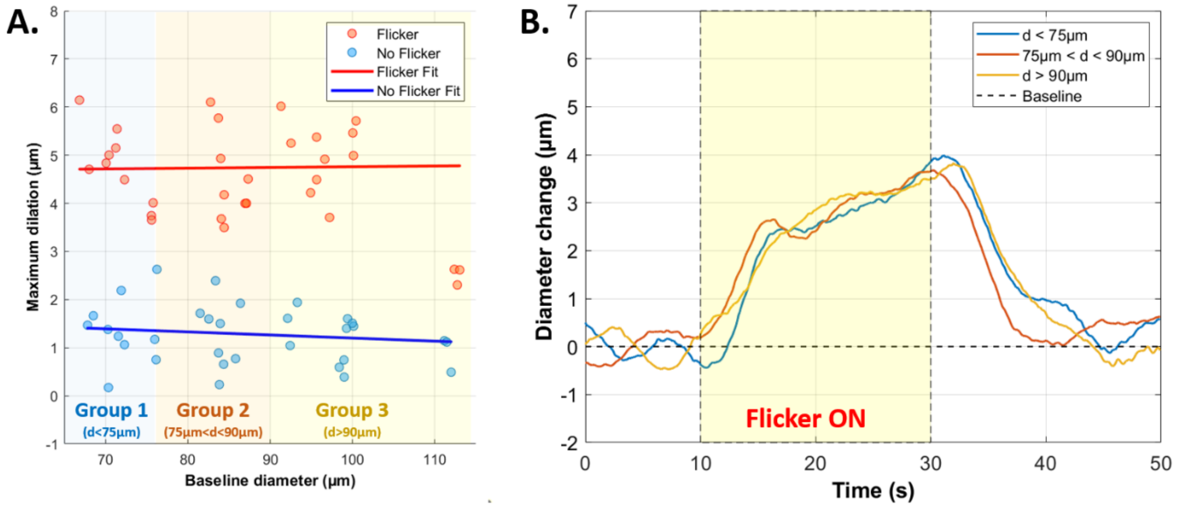

Figure S10: Influence of arterial diameter on the absolute maximum dilation after flicker-induced vasodilation. A) Absolute maximum dilation for each flicker stimulation (red) and no flicker acquisition (blue) as a function of baseline vessel diameter. B) Averaged absolute diameter change graph of every flicker test grouped by diameter “d”: blue: < 75 µm, red: between 75µm and 90µm, yellow: >90µm.

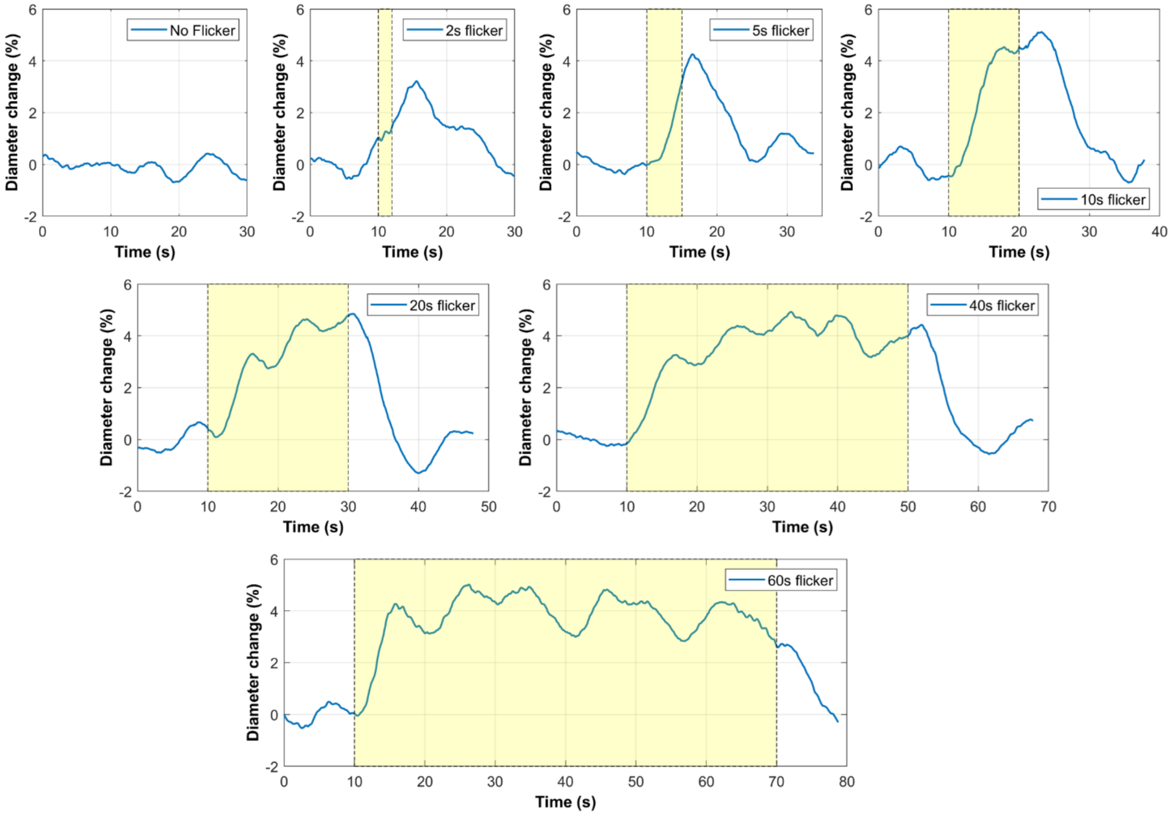

Figure S11: Study of the influence of flicker duration on NVC response. Respectively flicker duration of 0s, 2s, 5s, 10s, 20s, 40s, 60s.

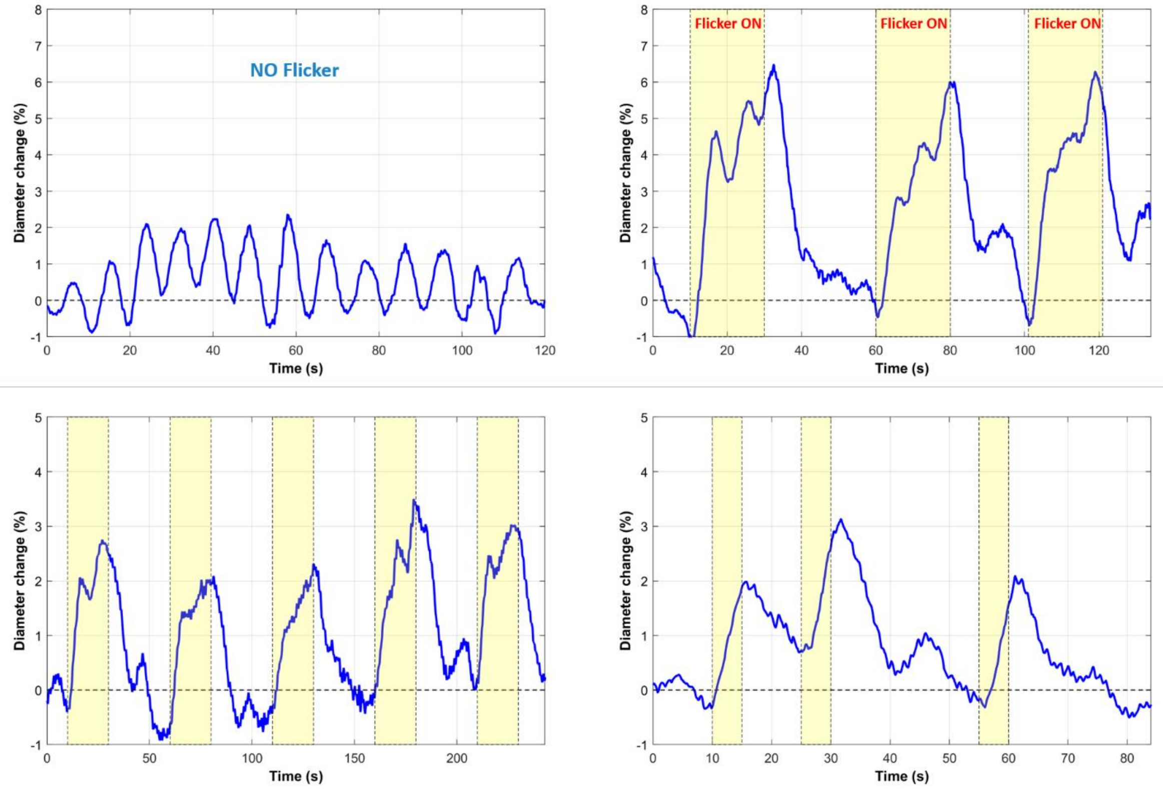

Figure S12: Study of the effect of consecutive flicker tests on the NVC response. Top left: No flicker measurement during 2min. Top right: Three flicker tests of 20s duration. Bottom left: Five flicker tests over 4 min. Bottom right: Three flicker tests of 10s with only 20s and 40s pause between flickers, highlighting an accumulative effect.

Datasets:

D1: “Data_for_Figs_2_and_3.mat” Data used to generate Figures 2 and 3 of the manuscript. Diameter change (%) over time with and without flicker stimulation for eight subjects.

D2: “Data_for_Fig_4.mat” Data used to generate Figure 4 of the manuscript. Diameter change (%) over time with and without flicker stimulation for eight subjects.

D3: “Data_for_Fig_5.mat” Data used to generate Figure 5 of the manuscript. Diameter change (%) over time for different flicker duration (three acquisitions for each scenario).

D4: “Data_for_Fig_6.mat” Data used to generate Figure 6 of the manuscript. Diameter change (%) over time with and without flicker stimulation for three different vessels over the same image field-of-view.
